# A Rational Approach to Sinus Augmentation: The Low Window Sinus Lift

**DOI:** 10.1155/2017/7610607

**Published:** 2017-02-26

**Authors:** Terry Zaniol, Alex Zaniol

**Affiliations:** Studio Dentistico Zaniol, Crocetta del Montello, Italy

## Abstract

Sinus augmentation is a well-known approach to treating alveolar bone ridge atrophy in the posterior maxilla. The preparation of the lateral window is crucial. Its size, design, and position in the vestibular sinus wall may affect the intra- and postsurgical complication rates and affect the intrasurgical activity of both surgeons and assistants. The present paper describes a rational technique that also exploits the guided surgery approach for design and preparation of a lateral window for sinus augmentation, the Low Window Sinus Lift. To illustrate the use of this approach, a case is presented in which the 50-year-old patient had the left maxillary first molar extracted, followed two months later by sinus augmentation and placement of three implants. One year after delivery of the definitive prosthesis, all three implants were successful, and the prosthesis was fully functional. Controlled studies should be undertaken to assess whether this technique provides significant advantages compared to other sinus augmentation approaches.

## 1. Introduction

Sinus augmentation is one of the most common bone-grafting surgeries, and its execution is within the reach of every oral surgeon with average skills [1]. Since its introduction by Tatum Jr. et al. [2, 3] and Boyne and James [4] in the 1980s, this surgical technique has been extensively studied. Widespread consensus [5, 6] about it has been reached, and it has been further refined [1, 7] to make it less invasive, spare the patient discomfort, and lower the rate of intra- and postsurgical complications. Along with the lateral approach originally proposed by Tatum Jr. et al., and Boyne and James, technique variants involving a crestal approach were later implemented by Summers [8] and other authors [7, 9–11]. The crestal approach, however, is not indicated if the residual bone ridge height is less than 4-5 mm [12]. In such cases, a lateral approach is still preferred.

A key element of lateral sinus lift surgery is designing and carrying out the lateral antrostomy. The design and position of the lateral window define the extent to which the mucoperiosteal flap must be elevated and affect the surgeon's subsequent actions. The window width, height, shape, and distance from the ridge border may have an impact on the angles that the sinus-membrane-elevation instruments must assume to effectively detach the membrane from the sinus floor. This, in turn, may affect the probability of membrane perforation, one of the most common complications [1, 13, 14]. Additionally, the extent to which the mucoperiosteal flap is elevated can limit easy access to the operatory field because of the need to keep the patient's vestibular tissues retracted for a longer time or to a wider amplitude, causing patient discomfort and operator fatigue.

To the authors' knowledge, no systematic investigation of the effect of the window design, size, and position on complication rates or effort required to carry out sinus lift surgeries has been carried out. Indications concerning the window size vary from author to author [15–21]. Different authors suggest that the lower antrostomy line should be positioned either flush with the sinus floor or up to 2-3 mm above it [1, 12, 22–24]. Analysis of the current clinical literature on lateral sinus lifts reveals great variability in the window shape, design, size, and position. Part of this variability is necessarily due to specific requirements arising from the individual patient anatomy [1], but part seems to depend only on the surgeon's personal habits. In the present paper, the authors propose a specific design for the lateral window based on rational considerations and observations. This design involves positioning the window as low and mesial as possible and has been named the “Low Window Sinus Lift technique.”

**Figure 1:**
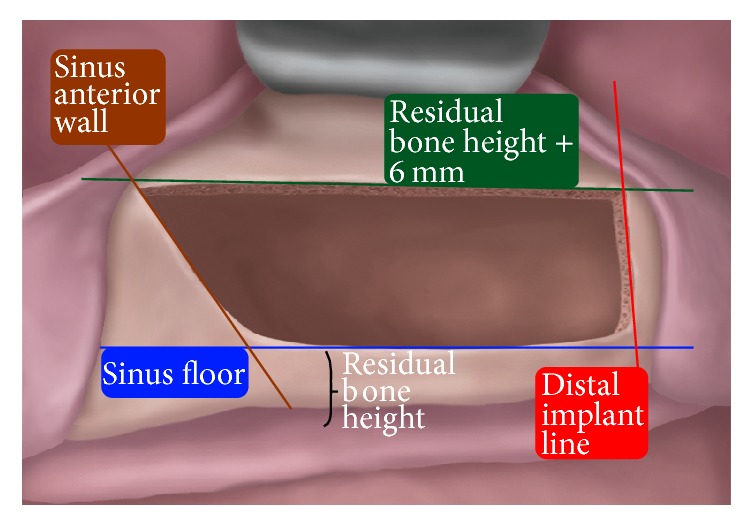
The Low Window Sinus Lift antrostomy. The lower osteotomy line (blue) is positioned flush with the sinus floor. The upper one (green) is 6 mm higher; that is, it is placed at a distance from the ridge equal to the residual bone height plus 6 mm. The mesial line (brown) is flush to the sinus anterior wall. The distal one (red) should be placed in correspondence with the position of the most distal implant.

The Low Window Sinus Lift technique involves designing the lateral window according to the scheme presented in Figure 1. The lower osteotomy line is always placed flush with the sinus floor. The window optimally should be 6 mm high. Accordingly, the cranial (apical) osteotomy line is positioned 6 mm above the sinus-floor level or, equivalently, at a distance from the ridge border equal to the residual bone height (RBH) plus 6 mm. The distal osteotomy line position varies corresponding to the most distal planned implant. Finally, the mesial osteotomy should be placed flush with the anterior wall of the sinus. Using this approach may allow preparation of the flap to be limited to a linear incision, one that preserves the attached gingiva of the most distal residual element present. Release incisions are not performed, and the mucoperiosteal flap may be elevated by a maximum of 10 mm.

The rationale for creating a low window at the most coronal and mesial possible position is that the more apical and distal the window is, the more difficult the surgical access to the sinus will be. Additionally, the position of the osteotomy lines provides specific surgical advantages. Placement of the lower horizontal osteotomy flush with the sinus floor eliminates any residual bone wall that could hinder detachment of the sinus membrane. The position of the distal osteotomy line is optimized according to the position of the most distal implant; extending it more distally than that provides no advantage and may result in elevation of a wider mucoperiosteal flap. Placing it more mesially forces the surgeon to detach a portion of the membrane in a “blind” condition, with no reference points. The position of the mesial osteotomy line, flush with the anterior sinus wall, allows for easier access to the sinus recess, that is, the zone where detaching the sinus membrane is usually more difficult. A window height of 6 mm is the minimum that allows for easy access of the membrane elevators. A smaller height would be an obstacle to membrane elevation, while a greater one would not provide any significant advantage but would require elevation of a wider mucoperiosteal flap [21]. An additional consideration is that when the maxillary ridge is more atrophic, the upper horizontal osteotomy will be lower (since the distance from the upper osteotomy to the sinus floor must be 6 mm) and less detachment of the mucoperiosteal flap will be required, reducing the overall invasiveness of the surgery.

The following case illustrates the use of the Low Window Sinus Lift technique.

## 2. Case Presentation

**Figure 2:**
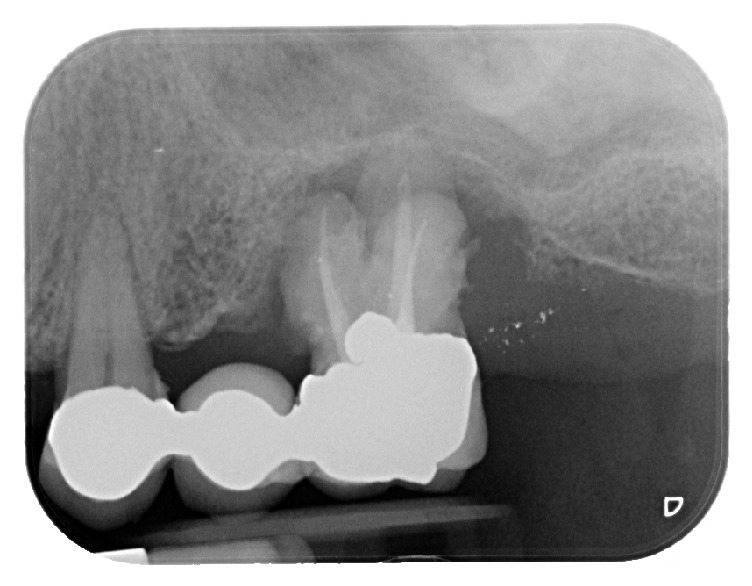
Intraoral radiograph at the patient presentation. Tooth 26 is affected by an endoperiodontal lesion and is lost.

**Figure 3:**
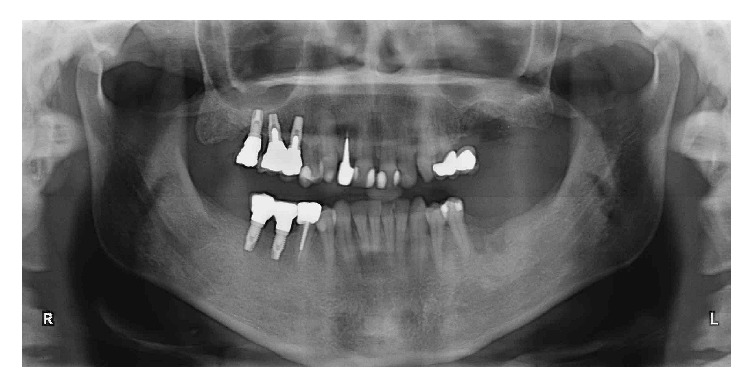
OPT recorded after sectioning the prosthesis and extracting the compromised element. The residual ridge presents a significant defect.

**Figure 4:**
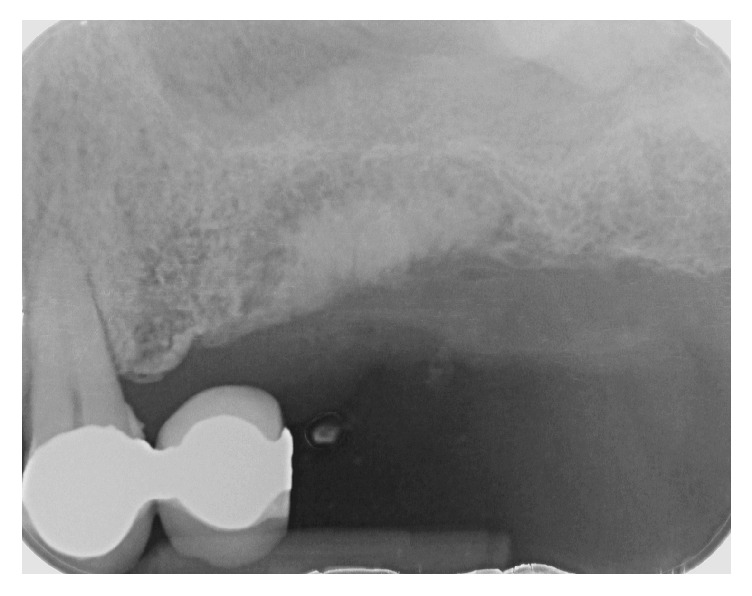
Intraoral radiograph collected two months later showing the limited thickness of the residual posterior ridge.

**Figure 5:**
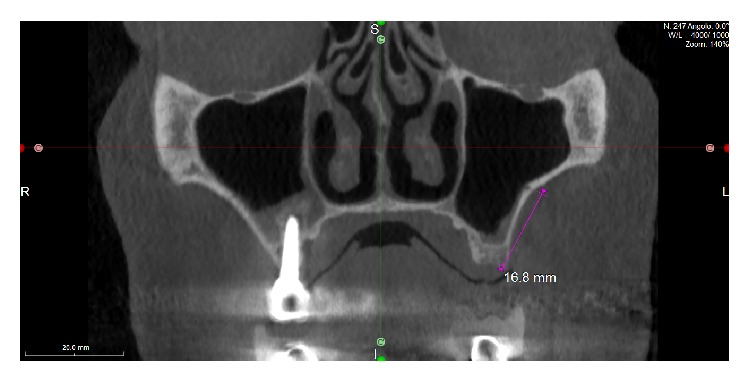
CBCT of the sinuses. The maxillary intraosseous anastomosis at the left sinus is 16.8 mm above the ridge coronal bone level.

**Figure 6:**
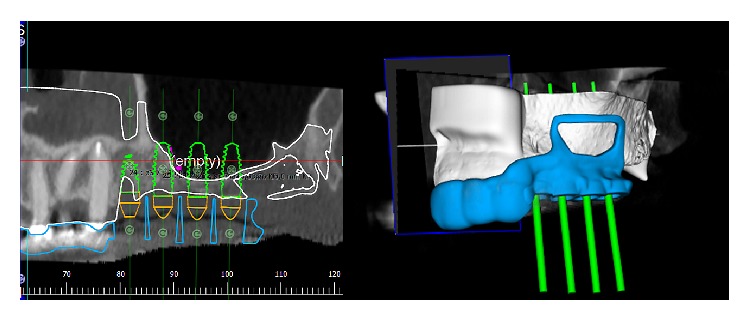
The position of the implants is preplanned on the CBCT scan. A surgical guide is designed that includes also the frame of the sinus antrostomy designed according to the low window principles.

The patient, a 50-year-old male, presented complaining about pain in his upper left maxilla that corresponded to earlier placement of a bridge connecting the first bicuspid to the first molar. The second bicuspid previously had been extracted. The patient underwent clinical examination and radiographic assessment, and the intraoral radiograph (Figure 2) showed an endoperiodontal lesion affecting the first molar. After sectioning the old prosthesis, the affected tooth was atraumatically extracted, and an orthopantomography (OPT) was collected (Figure 3). Two months later, the residual bone height was found to be insufficient to place osseointegrated implants (Figure 4). A CBCT examination was performed to evaluate the health and anatomical status of the left sinus (Figure 5). A rehabilitation plan involving sinus augmentation using the Low Window Sinus Lift approach and concomitant implant placement was developed, and the patient provided informed consent. The implant positioning was preplanned using the CBCT scan in order to have a surgical guide manufactured. The design of the surgical guide also included a guide for carrying out the lateral antrostomy according to the low window scheme previously described (Figure 6).

*Surgical Procedure*. Antibiotic prophylaxis (amoxicillin/clavulanic acid, Augmentin, Glaxo-SmithKline, Verona, Italy, 1 g 1 hour before surgery and then every 12 hours for 6 days) was initiated. The patient also was instructed to rinse with chlorhexidine 0.2% (Corsodyl, Glaxo-SmithKline) for two weeks after surgery. Ketoprofen 80 mg (Oki, Dompé, L'Aquila, Italy) was prescribed for pain as needed, but not to exceed every eight hours for seven days.

**Figure 7:**
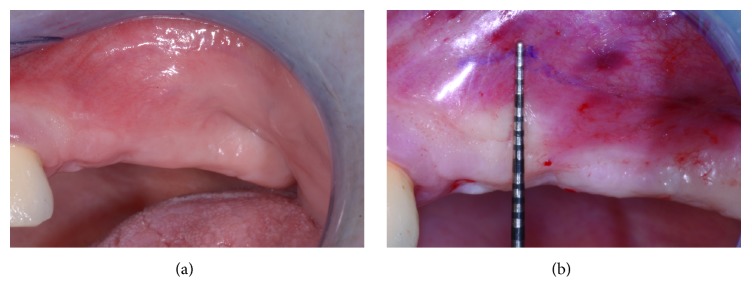
The clinical appearance of the edentulous posterior maxilla (a) and the flap design (b) at no more than 10 mm from the ridge.

**Figure 8:**
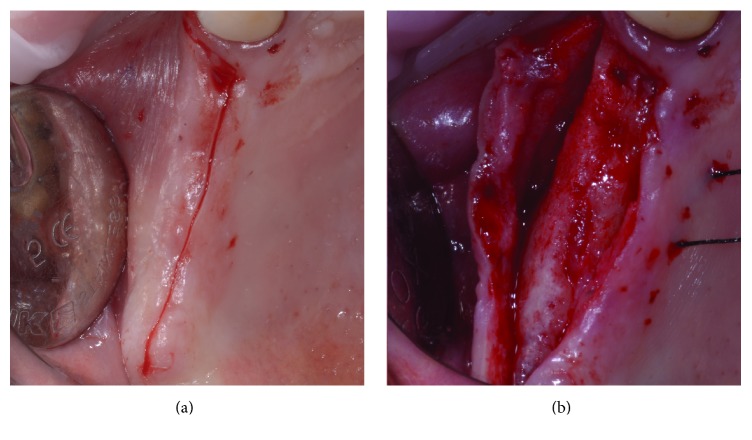
A single incision is performed on the medial, occlusal line of the ridge, preserving the papilla of the most distal residual element (a). No release incisions are carried out, and a full-thickness mucoperiosteal flap is elevated (b).

**Figure 9:**
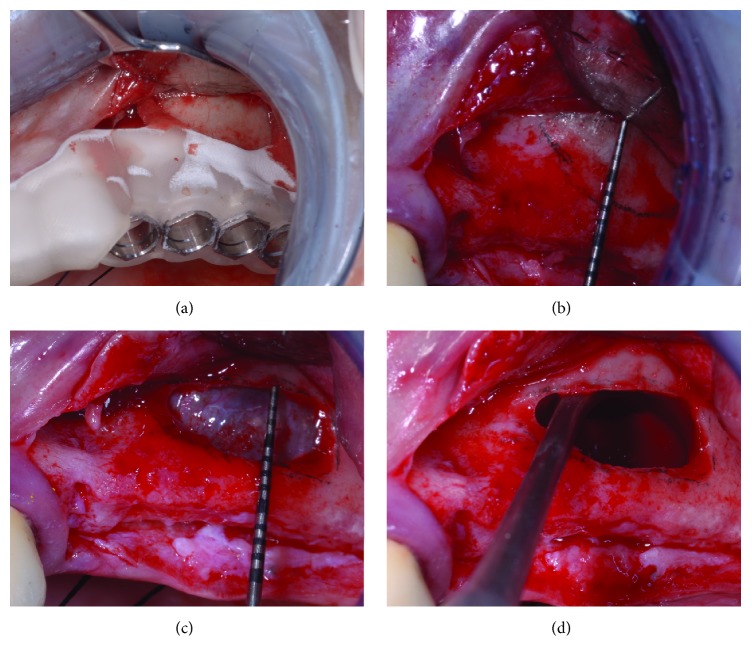
The preparation of the antrostomy and the elevation of the sinus membrane. First, the window is drawn on the vestibular bone wall with the aid of the surgical guide (a). The window is no more than 6 mm high (b). After performing the osteotomy (c), the sinus membrane is fully elevated (d).

**Figure 10:**
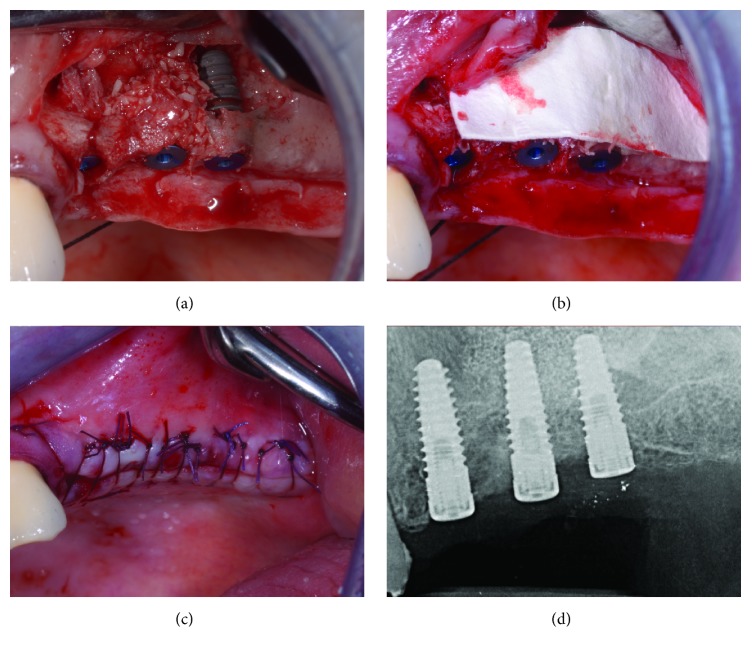
After partially filling the grafting site, implants are being placed with the aid of the surgical guide (a) and filling is complete (b). Implants are left submerged (c), and an intraoral control radiograph is collected (d).

To get easier access to the surgical area, a flexible aid (Optragate, Ivoclar Vivadent AG, Schaan, Liechtenstein) was placed. The surgical area was anesthetized with articaine hydrochloride 40 mg/mL with adrenaline 1 : 100,000. A full-thickness flap that enabled the apical osteotomy line to be drawn 10 mm above the ridge was elevated. Mesially, the incision was paramarginal to the more distal residual element in order to preserve its attached gingiva. No releasing incisions were performed either distally or mesially; that is, the incision had no vertical components (Figures 7 and 8). The access window was then drawn on the vestibular bone using a dermographic pencil and the surgical guide. Using standard piezoelectric tips under sterile saline irrigation, the window in the maxillary sinus lateral wall was then created. The sinus membrane was carefully elevated (Figure 9), and equine-derived cortical-cancellous granules, sized 0.5–1 mm (Osteoxenon, Bioteck, Arcugnano, Italy), were hydrated with sterile saline and inserted into the cavity, applying gentle pressure to stabilize them. Before the cavity was full, three 4.0 × 13.0 mm osseointegrated implants were placed in the positions indicated by the surgical guide. Filling of the cavity was then completed, and the mucoperiosteal flaps were sutured using nonresorbable 5.0 sutures (Figure 10).

**Figure 11:**
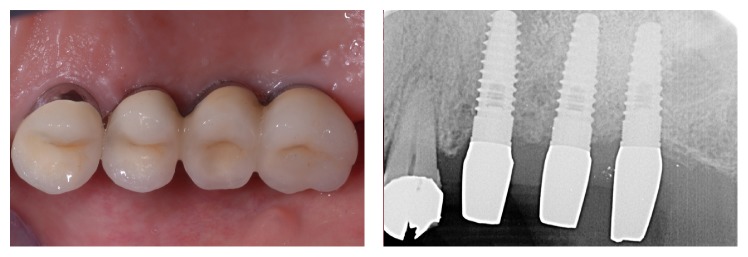
The final prosthetic rehabilitation.

**Figure 12:**
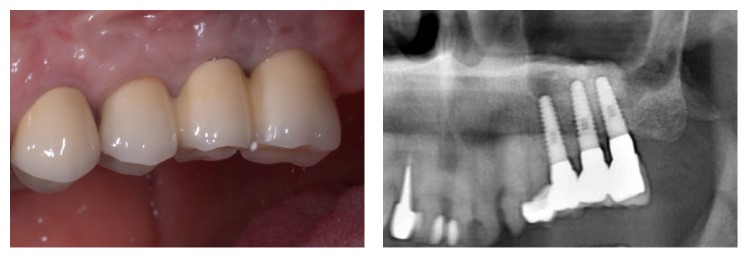
At the one-year control after definitive prosthetic rehabilitation, implants are successful and the prosthesis is fully functional.

The sutures were removed after 10 days. Six months after placement, the implants were uncovered, and healing screws were attached. Three weeks later, a radiograph was taken, and a dental impression was made using pick-up impression copings in order to manufacture a provisional prosthesis. This was delivered after 10 days, and the patient wore it for approximately three months, at which point the definitive abutments and metal-ceramic crowns were delivered (Figure 11). The maintenance program included professional oral hygiene at six and 12 months after rehabilitation with the definitive prosthesis (Figure 12).

No intraoperative or immediate postoperative complications occurred. The patient healed uneventfully. At the one-year follow-up, all implants were successful according to the criteria defined by Albrektsson et al. [25] concerning marginal bone levels changes. The prosthesis was fully functional (Figure 12). No significant differences were observed as far as the graft height stability was concerned; that is, the distance between the implant apices and the graft level showed no changes when compared to that at baseline (grafting surgery). The Full Mouth Plaque Score of the patient, which was 10% before surgery, had increased to 20% at the one-year control follow-up. The patient was therefore advised to take greater care of his oral hygiene at home, and more frequent hygiene treatments were planned. The patient was fully satisfied with his rehabilitation.

## 3. Discussion

The authors have used the Low Window Sinus Lift technique in more than 50 lateral sinus augmentations performed over the past four years. Invasiveness appears to be reduced because a smaller flap is usually necessary than with other lateral antrostomy preparations. Consequently, patients may experience less discomfort and fewer postsurgical complications, such as swelling or pain. The approach has also been surgeon-friendly, with access to the surgical site gained more easily. This in turn often reduces the need for the surgical assistant to provide retraction of lips and cheeks during the surgery. As in the case described, a flexible aid alone may provide sufficient retraction. Because of the low window position, the cortical layer that must be removed tends to be thinner. Osteotomy preparation thus tends to require less time. Moreover, detaching the sinus membrane tends to be accomplished more easily because, given the lower window position, the elevating movement occurs not only laterally but also upward. Together with the greater membrane visibility, these features may also reduce the intraoperative complications (e.g., sinus membrane tearing). Last but not least, positioning the window according to this technique would usually prevent the clinician from encountering the posterior superior alveolar artery [26, 27], thus minimizing the risk of damaging it. The short operative time, reduced invasiveness, and lower risk of membrane tearing and/or superior alveolar artery damage are the possible advantages of this technique over other current approaches.

It should be noted that this technique is effective insofar as it exploits the accuracy that can be achieved by current CAD-CAM manufacturing systems; that is, the surgeon, in order to carry out the technique properly, will need to have a CT or a CBCT scan performed and a surgical guide manufactured. Yet, the surgical guide for implant insertion, modified to incorporate the window frame, can be easily created and used effectively because of the low window position. In contrast, a higher window position tends to hinder correct positioning of such a guide because of the inclination of the vestibular ridge.

The Low Window Sinus Lift technique does not influence other significant sinus augmentation variables, such as the volume of biomaterial required or the length of the implants to be placed, and it does not preclude the possibility of performing a concomitant vertical/horizontal ridge augmentation by guided bone regeneration if necessary. In this case, the flap design will still involve performing release incisions in order to allow suturing without residual tension even if the ridge volume has been augmented.

In the authors' experience, the Low Window Sinus Lift technique has no technique-specific contraindications. The use of the surgical guide also minimizes the risk of creating the lower osteotomy in too low position, that is, one that will cut into the residual crestal bone below the sinus membrane.

Finally, the Low Window Sinus Lift technique requires following specific and replicable operative indications in designing the lateral window. This implies that clinical studies could be designed to investigate its effectiveness at lowering intraoperative complications, postsurgical patient discomfort, or other outcomes of interest, with no bias due to different window designs and positions such as that observed in current studies of lateral sinus augmentation.

The Low Window Sinus Lift technique proposed in the present study appears to be a replicable, rational approach to sinus lift augmentation that may entail significant advantages for both patients and surgeons. Controlled studies should be undertaken to investigate whether this technique provides significant improvements over alternative sinus augmentation approaches.
